# The Elusive Search for Success: Defining and Measuring Implementation Outcomes in a Real-World Hospital Trial

**DOI:** 10.3389/fpubh.2019.00293

**Published:** 2019-10-18

**Authors:** Heather L. Shepherd, Liesbeth Geerligs, Phyllis Butow, Lindy Masya, Joanne Shaw, Melanie Price, Haryana M. Dhillon, Thomas F. Hack, Afaf Girgis, Tim Luckett, Melanie Lovell, Brian Kelly, Philip Beale, Peter Grimison, Tim Shaw, Rosalie Viney, Nicole M. Rankin

**Affiliations:** ^1^Psycho-Oncology Co-operative Research Group (PoCoG), University of Sydney, Sydney, NSW, Australia; ^2^Centre for Medical Psychology and Evidence-Based Decision-Making (CeMPED), University of Sydney, Sydney, NSW, Australia; ^3^Rady Faculty of Health Sciences, College of Nursing, University of Manitoba, Winnipeg, MB, Canada; ^4^Psychosocial Oncology & Cancer Nursing Research, St. Boniface Hospital Research Centre, Winnipeg, MB, Canada; ^5^Centre for Oncology Education and Research Translation (CONCERT), Ingham Institute for Applied Medical Research, South Western Sydney Clinical School, University of New South Wales, Sydney, NSW, Australia; ^6^Improving Palliative, Aged and Chronic Care Through Clinical Research and Translation (IMPACCT), Faculty of Health, University of Technology Sydney, Ultimo, NSW, Australia; ^7^HammondCare Northern Sydney, Greenwich, NSW, Australia; ^8^Northern Clinical School, University of Sydney, Sydney, NSW, Australia; ^9^School of Medicine and Public Health, University of Newcastle, Newcastle, NSW, Australia; ^10^Cancer Services for the Sydney Local Health District (Incorporating Royal Prince Alfred, Concord and Canterbury Hospitals), Sydney, NSW, Australia; ^11^Chris O'Brien Lifehouse, Camperdown, NSW, Australia; ^12^Sydney Medical School, University of Sydney, Sydney, NSW, Australia; ^13^Charles Perkins Centre, Faculty of Health Sciences, University of Sydney, Sydney, NSW, Australia; ^14^Centre for Health Economics Research and Evaluation, UTS Business School, University of Technology Sydney, Sydney, NSW, Australia; ^15^Faculty of Health Sciences, University of Sydney, Sydney, NSW, Australia

**Keywords:** implementation science, health services research, outcome measurement, methodology, psycho-oncology, clinical pathways

## Abstract

**Objective and Study Setting:** Research efforts to identify factors that influence successful implementation are growing. This paper describes methods of defining and measuring outcomes of implementation success, using a cluster randomized controlled trial with 12 cancer services in Australia comparing the effectiveness of implementation strategies to support adherence to the Australian Clinical Pathway for the Screening, Assessment and Management of Anxiety and Depression in Adult Cancer Patients (ADAPT CP).

**Study Design and Methods:** Using the StaRI guidelines, a process evaluation was planned to explore participant experience of the ADAPT CP, resources and implementation strategies according to the Implementation Outcomes Framework. This study focused on identifying measurable outcome criteria, prior to data collection for the trial, which is currently in progress.

**Principal Findings:** We translated each implementation outcome into clearly defined and measurable criteria, noting whether each addressed the ADAPT CP, resources or implementation strategies, or a combination of the three. A consensus process defined measures for the primary outcome (adherence) and secondary (implementation) outcomes; this process included literature review, discussion and clear measurement parameters. Based on our experience, we present an approach that could be used as a guide for other researchers and clinicians seeking to define success in their work.

**Conclusions:** Defining and operationalizing success in real-world implementation yields a range of methodological challenges and complexities that may be overcome by iterative review and engagement with end users. A clear understanding of how outcomes are defined and measured, based on a strong theoretical framework, is crucial to meaningful measurement and outcomes. The conceptual approach described in this article could be generalized for use in other studies.

**Trial Registration:** The ADAPT Program to support the management of anxiety and depression in adult cancer patients: a cluster randomized trial to evaluate different implementation strategies of the Clinical Pathway for Screening, Assessment and Management of Anxiety and Depression in Adult Cancer Patients was prospectively registered with the Australian New Zealand Clinical Trials Registry Registration Number: ACTRN12617000411347.

## Introduction

Implementation science is defined as “the scientific study of methods to promote the systematic uptake of research findings and other evidence-based practices into routine practice, and, hence, to improve the quality and effectiveness of health services. It includes the study of influences on healthcare professional and organizational behavior” (1). Clinical pathways, increasingly used to inform patient management and care, provide evidence-based recommendations to guide best practice and consistent care for specific patient concerns in homogeneous patient groups. Whilst carefully designed evidence-based clinical pathways have demonstrated success in bringing about change in patient management (2) and improved patient outcomes (3, 4), they are not always successfully implemented and hence rarely lead to the desired real world practice change (5, 6).

The discipline of implementation science has grown considerably over the past decade, focusing on informing effective research translation into practice, through study of strategies which support the uptake of evidence-based interventions, and understanding of barriers impeding this process (1). Implementation research acknowledges that awareness of context and use of appropriately tailored implementation strategies are critical to success (7). To enable evaluation of such strategies, clear and well-defined markers of success should be selected and operationalized prior to embarking on implementation.

A key issue delaying progress in the discipline is a lack of clarity in defining the “success” against which strategies are judged. Defining success for clinical pathways in particular is recognized as complex, given they are comprised of multiple interacting components (8), each of which may require its own definition of success. A recent systematic review of guideline dissemination and implementation strategies (9) noted that definitions of effectiveness used to date are diverse, rarely explained in detail or grounded in theory, and often confound intervention and implementation success. They also tend to be categorical rather than continuous, meaning that findings are hard to interpret and cannot be generalized or made useful to practitioners and policy makers.

The Standards for Reporting in Implementation studies (StaRI) guidelines (10) facilitate clarity of reporting, thereby overcoming some of these issues. These guidelines highlight the importance of separating intervention success (e.g., treatment effectiveness) from implementation success (successful roll-out) to distinguish whether failure occurred due to the intervention not working (intervention failure), or whether the intervention was not implemented effectively including lack of uptake (implementation failure) (11).

To assist researchers in defining and reporting specific success outcomes, various evaluation frameworks have been developed (12), including the implementation outcomes taxonomy by Proctor et al. (11), which proposes eight distinct outcomes: acceptability, adoption, appropriateness, feasibility, fidelity, implementation cost, penetration and sustainability. Whilst these outcomes provide a working model for how to conceptualize success, they must still be operationalized via practical tools and measures. Also, collection of success data for real-world implementation research poses a challenge in busy, time-poor settings, where research is not the primary focus, even for engaged stakeholders (13–15). Thus, pragmatically, success measures should be brief, broadly applicable and sensitive to change (16). Currently there is little research that provides worked examples of how researchers and clinicians can respond to these challenges, engaging with the complex issues of defining and operationalizing success.

## Objective

The aim of this paper is to provide a conceptual approach to developing a clear definition and criteria for implementation success in a current cluster randomized controlled trial (ADAPT Cluster RCT); data collection and final results for this trial will be complete in 2020. The ADAPT Cluster RCT compares the effectiveness of implementation strategies to influence adherence to the Australian Clinical Pathway for the Screening, Assessment and Management of Anxiety and Depression in Adult Cancer Patients (ADAPT CP) (17). In line with StaRI guidelines, we specify the implementation strategies designed by the ADAPT Program Measurement and Implementation Working Group to support implementation, how they are anticipated to work, and how their success will be assessed in relation to pre-defined outcomes. To do this, we focus specifically on the StaRI reporting standards around methods of evaluation, which encourage researchers to report “defined, pre-specified primary and other outcomes of the implementation strategy and how they were assessed” (10). Using the ADAPT Cluster RCT as an example, and specifically the methodological work in designing and developing measurable outcomes for this trial, we illustrate how researchers can define implementation success when designing and developing implementation studies with guidance to translate measurement concepts into measurable data.

## Project Background

Full descriptions of the ADAPT CP (17, 18) and the ADAPT Cluster RCT protocol (19) are reported elsewhere but brief details are provided here to give context. The ADAPT Cluster RCT is registered with the Australian New Zealand Clinical Trials Registry ACTRN12617000411347. The ADAPT CP is designed to facilitate evidence-based responses to anxiety and depression in patients with cancer. It was developed to address research showing that, despite high rates of anxiety and depression in this population (3) and high acceptance that psychosocial care is integral to quality cancer care, anxiety and depression are often undetected, under-estimated and poorly managed in busy cancer services (20, 21). The ADAPT CP incorporates iterative screening and five steps of care, with specific recommendations for staffing, content and timing of interventions based on the level of anxiety and/or depression symptoms reported by the patient; and can be tailored to individual cancer services' available resources. Development was guided by existing empirical evidence and wide stakeholder consultation involving in-depth clinician interviews and a Delphi consensus process with >80 experienced multi-disciplinary clinicians working across a range of cancer services in Australia (18, 22). Implementation of the ADAPT CP by cancer services was anticipated to require significant change to workflow and resourcing.

Following a barriers and facilitators analysis (22) a range of evidence-based, online resources were developed to facilitate successful implementation of the ADAPT. Online resources include education for staff about how to discuss anxiety and depression with patients, explain screening and make referrals if necessary (hosted on eviQ, a portal for oncology health professional education hosted by the Cancer Institute NSW); information for patients about anxiety and depression; a self-guided cognitive-behavioral program (iCanADAPT) for anxiety and depression (23); and an individualized referral network map. These resources are accessible via the ADAPT Portal, an operational web-based system that facilitates staff and patient access to regular screening and the evidence-based step allocation and referral recommendations outlined in the ADAPT CP (24).

Development of implementation strategies for ADAPT were guided by the Promoting Action Research in Health Services (PARiHS) framework (25) (see Table 1). Within the ADAPT Cluster RCT, 12 cancer services (including approximately 2,000 patients), implementing the ADAPT CP are randomized to receive one of two different implementation support packages. As part of this large implementation research program, we designed a cluster randomized trial, the ADAPT Cluster RCT, to evaluate the level of implementation support required (core vs. enhanced) to achieve adherence to the ADAPT CP over a 12-month period.

**Table 1:** Implementation strategies.

	**Both core and enhanced implementation strategies arms**	**Enhanced implementation strategies arm *only***
**Strategy**		
Awareness campaign	•Baseline roadshow, posters, email from site champion to all staff	•Additional posters and newsletters during implementation
Champions	•Clinical, administrative, management	•Additional monthly proactive contact with Champions during implementation
Staff training	•Portal Training + user guides •Clinical Pathway Training	•Refresher training as required
Academic detailing and support	•Baseline written report: staff readiness •Tailoring of ADAPT portal to site •Study close meeting	•Verbal reports with discussion •Quarterly review of portal
Reporting	•Monthly written reports on portal statistics	•Verbal reports with discussion
Technological support	•IT support for the ADAPT portal	

## Operationalizing Success in the ADAPT Cluster RCT

In selecting and defining outcomes of implementation success for the ADAPT Cluster RCT, we were informed by the Implementation Outcomes framework and the StaRI Statement and Checklist (10, 11). Both documents propose that their frameworks be used as a catalyst for discussing and defining how implementation studies are conceived, planned, and reported. These documents prompted discussion within the ADAPT Steering Committee and a smaller working group with specialized interest and expertise in measurement of success, around the structure and collection of implementation outcome data.

To capture the dynamic nature of success outcomes, baseline, mid-point and endpoint data collection using questionnaires and semi-structured interviews was planned. Thus, data collection occurs just after staff have been trained in the ADAPT CP and associated resources and been exposed to some implementation strategies (T0), and again after staff have experience of the pathway in practice, as well as the full suite of implementation strategies at 6 and 12 months following implementation (T1 and T2).

## Measuring Success of the ADAPT CP Implementation, The Intervention Components and the Implementation Strategies

A major challenge in the development of implementation outcomes for the ADAPT Cluster RCT, and the subject of this methodology paper was differentiating between the three concepts underlying the implementation: the ADAPT CP; the intervention components, and; the implementations strategies. To address this, we describe how the ADAPT Program investigators, led by the Measurement and Implementation Working Group membership agreed to measure successful implementation of the ADAPT Clinical Pathway, and then agreed on methods of measuring success of the intervention components and implementation strategies, with the goal of choosing theoretically, empirically and psychometrically rigorous measurement approaches that matched implementation goals and frameworks.

### Primary Outcome

#### Determining Success of the ADAPT CP Implementation

Based on a comprehensive literature review and iterative discussion within the Measurement and Implementation Working Group, the primary outcome agreed on for the ADAPT Cluster RCT was site adherence to the key tasks of screening, assessment, referral and management defined by the ADAPT CP. Much of the existing research addresses medication adherence and is therefore focused on algorithms describing patient medication intake. Such outcomes are rigid, clearly defined and easily measurable. Measuring adherence to a clinical pathway is more complex as it is a multifaceted and multi-disciplinary therapeutic intervention. To address this issue, we defined adherence as the delivery, *by any appropriate staff* (individually defined at each site), of the main components of the ADAPT CP.

The ADAPT CP uses a stepped care model, allocating patients based on their screening scores and, if required, a triage conversation with a clinical staff member, into one of five steps, from minimal to very severe level of anxiety and/or depression, each with its own recommended treatment regimen. An added complexity is the need to consider individual differences between patients, meaning that the relevant recommendations of the ADAPT CP may differ even within patients allocated to the same step. To address this complexity, we specified adherence as the percentage of all actions *appropriate to the patient's level of anxiety and depression as confirmed by the triage conversation*, undertaken for each patient at each screening episode. Quantitative data collected via the ADAPT Portal enables capture of all adherence data.

To provide a more clinically relevant measure of adherence, we further defined a categorical outcome (adherent: ≥70% of patients experience ≥70% of key components recommended by the ADAPT CP (e.g., screening, triage, referral and re-screening); or non-adherent: <70% of patients experiencing ≥70% of key components recommended by the ADAPT CP), based on accepted implementation targets (26, 27).

### Secondary Outcome

#### Determining Success of the Intervention Components and Implementation Strategies to Support Implementation of the ADAPT CP

Using the StaRI guidelines, a process evaluation was planned to explore participant experience of the ADAPT CP, resources and implementation strategies according to the Proctor Implementation Outcomes framework. We translated each outcome into clearly defined and measurable criteria for our study outcomes (Table 2), noting whether each addressed the ADAPT CP, resources or implementation strategies, or a combination of the three. We applied the Proctor definitions by inserting the context, the relevant participants from whom data was being collected. The expert working group agreed that this step would clarify the data and outcomes to support reporting of the ADAPT cluster RCT findings. Table 2 also indicates the agreed timepoints of data collection prior to the 12 month supported implementation period (T0), at 6 months (T1) and at 12 months (T2) following implementation to add to the evidence on how implementation outcomes may shift over time, potentially allowing research to identify “leading” and “lagging” indicators of success (11).

**Table 2:** Success outcomes based on Proctor et al. (11) as defined for the ADAPT RCT.

**Outcome**	**Definition from Proctor et al. (11)**	**Definition as applied to ADAPT Cluster RCT**	**Timing of measurement**	**Measurement source**
1, Acceptability	The perception among implementation stakeholders that a given treatment, service, practice, or innovation is agreeable, palatable, or satisfactory	Cancer staff perceptions of *ADAPT CP, intervention components and implementation strategies* as agreeable, palatable, or satisfactory	T0: Expected T1 and T2: Experienced	*Staff questionnaire:* Hands4U (28) and additional tailored questions
2. Adoption	The intention, initial decision, or action to try or employ an innovation or evidence-based practice	The intention and uptake of the ADAPT Resources and the Clinical Pathway by cancer services, cancer service staff and patients	T0: Expected T1 and T2: Experienced	*Staff questionnaire:* Organizational Readiness for Implementing Change (ORIC) (29), Hands4U (28) and additional tailored questions *Semi-structured interviews ADAPT team contact log*
3. Appropriateness	The perceived fit, relevance, or compatibility of the innovation or evidence-based practice for a given practice setting, provider, or consumer; and/or perceived fit of the innovation to address a particular issue or problem	The extent to which cancer staff believe that *ADAPT CP and resources* have fit, relevance and compatibility at the level of their setting, their role, and the needs of their patients	T0: Expected T1 and T2: Experienced	*Staff questionnaire:* Organizational Readiness for Implementing Change (ORIC) (29), Hands4U (28) and additional tailored questions *Semi-structured interviews*
4. Feasibility	The extent to which a new treatment, or an innovation, can be successfully used or carried out within a given agency or setting	The extent to which implementation *of ADAPT CP, resources and implementation strategie*s is considered feasible for each service, given their different needs and resources, allowing for individual tailoring	T0, T1, and T2	*ADAPT team contact log Implementation Checklist & Observational Diary Site Profile Staff questionnaire:* Organizational Readiness for Implementing Change (ORIC) (29), Hands4U (28) and additional tailored questions *Semi-structured interviews*
5. Fidelity	The degree to which an intervention was implemented as it was prescribed in the original protocol or as it was intended by the program developers	The degree to which each service receives the *implementation strategies* as planned within their randomization (core vs. enhanced)	T1, T2, and T3	*ADAPT team contact log Implementation Checklist & Observational Diary Audit data* from ADAPT Portal
6. Implementation cost	The cost impact of an implementation effort, which is dependent on the cost of the intervention components, the cost of the implementation strategies used to implement and the costs of delivery within the particular setting	The cost of *ADAPT CP, resources and implementation strategies*, across different services (acknowledging that the costs of implementation for different services will vary)	T3	*ADAPT team contact log Medical Benefit Scheme (MBS) and Pharmaceutical Benefit Scheme (PBS) data*
7. Penetration	The integration of a practice within a service setting and its subsystems	The extent to which *ADAPT CP, resources and implementation strategies* were used by the cancer service and referral networks	T1 and T2	*Audit data* from ADAPT Portal *Implementation Checklist & Observational Diary Audit data* from EviQ
8. Sustainability	The extent to which a newly implemented treatment is maintained or institutionalized within a service setting's ongoing, stable process of operation	The extent to which *ADAPT CP and resources* become institutionalized as part of routine standard care service delivery as indicated by their continued use after the ADAPT RCT ends	T3	*Audit data* from ADAPT Portal *Implementation Checklist & Observational Diary Audit data* from EviQ

*T0, baseline; T1, implementation midpoint (6 months); T2, implementation endpoint (12 months); T3, final audit data collation (3 months post implementation endpoint)*.

A rapid review of the literature identified measures of success with strong psychometric properties and face validity that mapped well to the PARiHS domains, and that were concise to minimize participant burden. We chose the Organizational Readiness for Implementing Change (ORIC) (29) (measuring staff perceptions of organizational confidence, commitment and motivation to adopt ADAPT CP) and the Hands4U questionnaire (30), tailored for the purposes of the ADAPT CP (measuring staff personal confidence in implementing ADAPT CP, attitudes, intention, satisfaction regarding ADAPT CP, the resources and implementation strategies and perceived peer and leadership support for ADAPT CP).

After mapping these measures onto the eight outcomes described above, we identified gaps in the selected validated measures where important outcomes in the context of implementation of the ADAPT CP remained, including: perceived need, belief in the evidence base, perceived strength of leadership support, credibility of the research team and organizational leaders, perceived workload/burden of implementing, organizational fit and benefit and the organizational burden/costs. We developed 13 study-specific items to address these gaps, listed in Table 3.

**Table 3:** Mapping questionnaire items to implementation outcomes.

**Acceptability** •People feel confident that the organization can get people invested in implementing the anxiety and depression pathway (ORIC) •People are committed to implementing the anxiety and depression pathway ORIC •People want to implement the anxiety and depression pathway ORIC •People are motivated to implement the anxiety and depression pathway ORIC •High quality evidence that psychological interventions can reduce anxiety or depression in cancer patients (Additional) •The team evaluating the implementation of the anxiety and depression pathway have high credibility (Additional) •People understand why the organization needs to implement the clinical pathway (Additional) •The ADAPT CP aligns with our organization's mission and goals (Additional) •It is important to identify anxiety and depression in cancer patients (Hands4U)
**Adoption** •People here are determined to implement the anxiety and depression pathway (ORIC) •I will probably use the anxiety and depression pathway (Hands4U) •I will probably use a screening tool to identify anxiety and/or depression in cancer patients (Hands4U) •I will probably use the ADAPT Portal (Hands4U) •People who work here will do whatever it takes to implement the anxiety and depression pathway (ORIC)
**Appropriateness** •People feel confident that the organization can get people invested in implementing the anxiety and depression pathway (ORIC) •People who work here will do whatever it takes to implement the anxiety and depression pathway (ORIC) •People who work here feel confident that the organization can support people as they adjust to implementing the anxiety and depression pathway (ORIC) •Patients in our local service would benefit from treatment for anxiety and/or depression (Additional) •High quality evidence that psychological interventions can reduce anxiety or depression in cancer patients (Additional) •People understand why the organization needs to implement the clinical pathway (Additional) •The ADAPT CP aligns with our organization's mission and goals (Additional) •It is important to identify anxiety and depression in cancer patients (Hands4U)
**Feasibility** •People who work here feel confident that the organization can support people as they adjust to implementing the anxiety and depression pathway (ORIC) •People who work here feel confident that they can handle the challenges that might arise in implementing the anxiety and depression pathway (ORIC) •People who work here feel confident that they can coordinate tasks so that implementation goes smoothly (ORIC) •People who work here feel confident that they can manage the politics of implementing the anxiety and depression pathway (ORIC) •Implementing the anxiety and depression pathway will increase my workload (Additional) •Implementing the anxiety and depression pathway will take up too much of my time (Additional) •Implementing the anxiety and depression pathway will cost the organization too much money (Additional) •I am confident we have the necessary staff to implement the anxiety and depression pathway (Additional) •I am confident we have enough resources to implement the anxiety and depression pathway (Additional) •I am satisfied with the opportunities made available to me to use the ADAPT CP and the ADAPT Portal. (Hands4U)
**Fidelity** •People who work here feel confident that they can keep track of progress in implementing the anxiety and depression pathway (ORIC)

We also included in design the use of study logs and checklists to document interactions between the research team and study sites, combined with semi-structured interviews with multidisciplinary service staff involved in the implementation of ADAPT to complement the quantitative survey data to gather the most holistic impression of success. The semi-structured interviews comprise questions designed to assess all elements of the implementation process. Creation of the interview guide was informed by the PARiHS framework, the Consolidated Framework for Implementing Research [CFIR (31)] and a recent systematic review of hospital-based implementation barriers and facilitators (32). It includes questions both about specific components of ADAPT CP and more general insights into each service context. It was pilot tested by two members of the research team (LG and PB).

## Operationalizing Proctor's Implementation Outcomes for Success

We describe how each of the eight implementation outcomes is applied within the ADAPT Cluster RCT, demonstrating how quantitative and qualitative data are aligned to each outcome.

### Acceptability

Proctor and colleagues note that assessment of acceptability must focus on the stakeholder's “knowledge of or direct experience with various dimensions of the treatment to be implemented, such as its content, complexity, or comfort” (p. 67), ascertained via formal, objective data-collection of stakeholder views. Quantitatively, we operationalized acceptability with the questions (see Table 3) covering confidence, commitment, motivation, credibility, evidence, perceived importance and alignment with service or institutional goals.

Qualitatively, interview questions probe attitudes toward the ADAPT CP, the resources and strategies. Questions address perceptions regarding: the utility, helpfulness and likely efficacy of the ADAPT CP; service attitudes toward research utilization, quality improvement (QI), professional development, new initiatives, mental health and technology; whether services support staff with time and resources to implement new initiatives; staff receptivity to change; service attitudes to implementation strategies; and perceived role of champions and leadership within services.

Mid-way, and at the end of the implementation phase (T1 and T2), additional interview questions explore experience of the ADAPT CP implementation and the implementation strategies to date, positive or negative elements of these, whether strategies and resources are a suitable fit for the service and the staff profile, and whether strategies occur as planned.

### Adoption

The implementation of a multi-faceted intervention like a clinical pathway means that adoption must be considered in relation to both the intervention and the implementation process, the dual strands highlighted by the StaRI checklist. In considering adoption, we sought to differentiate between adoption of the clinical pathway, the intervention components and implementation strategies and decided to measure adoption of the implementation strategies separately as part of feasibility and fidelity.

In line with the StaRI checklist (10), we differentiated between adoption of the ADAPT CP and the intervention components, noting that the distinction between these dual strands is not always clear cut but can aid in clarity of study design and reporting (11).

We decided that adoption of the ADAPT CP was covered by the primary outcome of adherence. At baseline, we supplement this with questionnaire items assessing *intention* to use the CP (Table 3).

Adoption of ADAPT resources are measured by quantifying staff training user numbers, page hits on referral templates within the ADAPT portal and patient user numbers of the online therapy program, iCanADAPT, and anxiety/depression resources.

Adoption of ADAPT implementation strategies is measured quantitatively via page hits on system generated reports, ADAPT logs of staff attendance at meetings and training sessions, and email/phone contacts with each site regarding IT and training.

While the above measures are at organizational level using data from the ADAPT Portal and online registration numbers, qualitative data assesses adoption of implementation strategies at the individual level, ensuring that levels of adoption are measured at the staff and organizational level (10). Qualitatively, interview questions probe barriers and facilitators to staff engagement, whether implementation strategies are actioned, which strategies are most or least helpful and how they enable implementation.

### Appropriateness

“Appropriateness” and “acceptability” are often used interchangeably in the literature; however, a distinction between these two constructs is crucial. Proctor et al. suggest that appropriateness encompasses provider perceptions of the fit of a program to their service (including mission or values), their role or skillset, as well as fit to the patient population. They also note that such perceptions may be shaped by the organizational culture. Assessment of this outcome requires recognition and understanding of the site context and its role in shaping provider perceptions. We assess appropriateness at the organizational level via both staff interviews as well as field observations made by the research team.

Quantitatively, we operationalized appropriateness with the questions covering organizational fit, confidence, commitment, motivation, evidence, perceived benefit, importance and alignment with service or institutional goals (Table 3).

Qualitatively, appropriateness is assessed with interview questions probing whether the ADAPT CP, the resources and strategies are needed, would work within the service, and would fit with a service's mission and values.

### Feasibility

Proctor et al. note that an intervention may be appropriate for a service in terms of fit to values and goals, but not feasible due to lack of resources or infrastructure.

Quantitatively we assess feasibility of the ADAPT CP with questionnaire items covering confidence, perception of service capacity and capability (Table 3).

Similarly to appropriateness, feasibility has much to do with our understanding of the environment and resources (staff profile and workload). We capture current staffing numbers and overall staff profile supporting each cancer service during the engagement period with each service and reflect on any impact on the ability to implement the ADAPT CP, deliver and respond to implementation strategies.

Qualitatively, at all timepoints we assess feasibility through questions related to barriers and facilitators to the ADAPT CP and the implementation strategies in practice, and how or if they are overcome.

### Fidelity

Fidelity is defined as the degree to which an intervention was implemented as it was prescribed in the protocol or as intended by the program developers (11). Fidelity to the intervention is measured in efficacy and effectiveness trials, yet there are few instruments for measuring fidelity to implementation. It is typically measured by comparing the original evidence-based intervention and the disseminated/implemented intervention in terms of factors such as adherence, quality of delivery, program component differentiation, exposure to the intervention, and participant responsiveness or involvement (26, 27, 33).

Defining success is challenging for flexible interventions that allow local adaptation in order to increase their relevance and applicability (34–36), and which respond reflexively to unique characteristics and unpredictable reactions in their intervention settings (37). The “*fidelity/adaptation dilemma*” (38) and its resolution is regarded as one of the most important challenges for evaluation (39). The StaRI guidelines highlight the important distinction between active or core components of an intervention (to which fidelity is expected), and modifiable components, which may be adapted by local sites to aid implementation.

We defined non-modifiable components of the ADAPT CP and implementation strategies, as well as those that could (and should) be tailored to individual site requirements (see Table 4). Within the ADAPT Cluster RCT, we are able to apply fidelity first to the implementation of the ADAPT CP, measured by adherence to the recommendations (screening, triage, step allocation and intervention where appropriate), and second, to the level of implementation strategies received as planned according to randomization (core vs. enhanced). The primary data source for assessing fidelity to the ADAPT CP is the captured data points from the ADAPT Portal which contribute to the primary outcome of adherence. Content of key information in online education and therapy programs were not flexible and fidelity data are captured during delivery.

**Table 4:** Fixed or flexible components and strategies.

**Intervention components**	**Details**	
ADAPT portal	(online screening, triage, referral management)	Fixed components—Tailoring permitted
Patient information		Fixed
Health professional education (eviQ)		Fixed
iCanADAPT online therapy program		Fixed
**Implementation Strategies**	**Details**	
Awareness campaign	Roadshow Poster Campaigns Emails Newsletters	Flexibility (tailored content)
Champions	Clinical, Administrative, Management	Fixed
Staff training	Portal Training + user guides Clinical Pathway Training Health Professional Training	Flexibility (tailoring to available scheduling)
Academic detailing and support	Portal tailoring and local referral network and pathway mapping Audit and feedback—Face to face/email communication 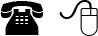 ADAPT Support Service	Fixed—tailoring permitted
Reporting	Automated report generation	Fixed
Technological support	ADAPT Portal	Fixed

Flexibility or tailoring was permitted in the *delivery* of the listed implementation strategies (Table 4), governed by the service scheduling (e.g., training), and tailored content for awareness campaigns to meet service priorities. Additional flexibility was permitted to respond to audit and feedback provided to participating services and to requests for further support throughout the implementation period. Assessment of these outcomes is challenging, as they do not fit well with the traditional method of measuring fidelity (checklists based on audio or video recordings, self-report, direct observation). Within the ADAPT Cluster RCT, the ADAPT team record the planned and actual delivery of the implementation strategies in the ADAPT Implementation Checklist and Observational Diary. ADAPT Contact logs are used by the ADAPT team delivering the implementation strategies to record any instances of tailoring or variation in non-modifiable aspects of both the CP and implementation strategies. Therefore, data from both the ADAPT Implementation Checklist and Observational Diary and ADAPT Contact log will be utilized to assess fidelity to the planned implementation strategies.

### Implementation Cost

Much cost research focuses on quantifying the cost of the intervention (40), while comparatively little data exists on the best ways to capture implementation costs (41). Direct measures of implementation cost are needed to inform decision-making on implementation strategies, yet implementation costs are challenging to measure, as they need to include billable costs of all resources used as well as administrative costs and employee time (42), particularly in the case of complex interventions with multi-component strategies. It is recognized that implementation costs will vary according to setting and there are many challenges involved in assessing the true cost. StaRI guidelines advocate the importance of separating implementation process costs from intervention costs at the design stage.

In the ADAPT Cluster RCT, we sought to address this issue by costing each implementation strategy and each intervention component. Implementation costs include direct costs, time spent at each site by the research team and opportunity costs. These include logging all contact with services, received and/or initiated by the ADAPT team, allocating this also to a planned (linked to implementation strategies listed in Table 1), or unplanned categorization to capture number of contacts with services throughout the implementation period. Time, type of contact, issue and related strategy are also collected, enabling a monetary cost to be calculated for staff time in supporting the ADAPT CP implementation, as well as costs associated with services provided and funded by the Australian Department of Health, via the Medicare Benefits Scheme from audit data, staff wages, and expenditure reports. These data will contribute to a final cost and value of implementing the ADAPT CP, with success being determined through health economic principles.

In the data collected from services, we determine staffing profile at the beginning of the implementation and at midpoint and endpoint. One question in our staff survey directly asks for agreement or not to the statement “*Implementing the anxiety and depression pathway will cost the organization too much money*.” These data will provide some contextual information which may support health economic analysis tapping into perceived value rather than monetary outlay.

### Penetration

To date, penetration has been captured in a range of ways by implementation scientists, from examining service recipients who received the desired care (43), to looking at the number of providers who delivered the desired intervention. Proctor et al. note that penetration is not frequently used in the literature as a term, but may have overlap with terms such as saturation, research or institutionalization.

In the ADAPT RCT, penetration is operationalized to mean the extent to which ADAPT resources and Clinical Pathway components are used by the cancer service and referral networks. This outcome is assessed quantitatively in surveys completed by staff at midpoint and endpoint where items ask whether staff are aware of implementation strategies (e.g., posters, newsletters, reports). Qualitatively, interview questions about penetration focus on staff engagement, whether staff follow the ADAPT CP in practice and use the resources, whether the experience is positive and therefore a good news story within and beyond the service or department, and whether the implementation strategies enable the implementation by bedding down new processes and practice.

Whilst integration is not an explicit outcome in the Implementation Outcomes framework, adoption, fidelity and penetration all provide valuable information from where integration into routine care in the case of the ADAPT CP can be extrapolated. Integration reflects the medium to longer-term outcomes of whether an intervention becomes an integrated component of standard care, while longer-term integration will be determined in outcomes measuring sustainability.

### Sustainability

Sustainability, the last aspect of the Proctor Implementation Outcome framework, is a term with varied meanings and interpretation (11). Proctor et al.'s definition incorporates key aspects of other theories, emphasizing program integration into organizational policy and practice and encouraging exploration of the processes through which a program becomes institutionalized. They note that penetration and sustainability are likely to be linked, as higher penetration of an intervention may contribute to long-term sustainability. To date, there has been greater focus on sustainability in conceptual rather than empirical papers. Where sustainability has been explored in trials, outcomes have focused on patient level changes, rather than measuring factors related to institutionalization in health services (44).

Within the ADAPT Cluster RCT, we defined sustainability in a way that would capture the extent of maintenance and institutionalization of the ADAPT CP within the service (as shown by the ADAPT Portal audit data at the end of the 12-month implementation period), as well as exploring stakeholder intent to maintain implementation of the ADAPT CP from qualitative data.

In terms of staff reported data, we have aligned one question in the Staff Survey to the theme of sustainability, namely: “People who work here feel confident that they can keep the momentum going in implementing the anxiety and depression pathway (ORIC).” Similar to themes we aligned with penetration, qualitative questions probing sustainability focus on staff engagement with the ADAPT CP in practice, their positive or negative experiences and, as a consequence, whether staff perceive the initiative continuing beyond the ADAPT Cluster RCT experience. Ideally, additional data collection for at least 12 months beyond the implementation period of services using the ADAPT Portal will provide more robust sustainability and integration data that can contribute to health services implementation. For the ADAPT Cluster RCT, this aspect can be predicted only based on service engagement over the ADAPT RCT 12 month supported implementation period, influenced or not by randomization to one of two different implementation packages.

## Discussion

In undertaking the process of defining success for the ADAPT Cluster RCT, it became clear that there is a paucity of research that explores and systematically recounts the ways in which outcomes for success are selected and operationalized. We noted the importance of specifying theoretically and contextually sensitive definitions of success outcomes and drew from both existing frameworks and ongoing consultation with key stakeholders and clinical service staff to tailor and revise our outcome definitions. Our attempt to separate outcomes, components and strategies described above in the ADAPT Cluster RCT organization and design has shown that measuring implementation success using existing frameworks was not straightforward. There is likely to be interaction across success outcomes, for example the impact of acceptability on adoption, penetration and sustainability. Therefore, using a conceptual approach to mapping the relationships between success outcomes may provide some valuable insights into this issue.

We have generated a combination of qualitative and quantitative measures to capture our primary and secondary success outcomes, to capture both the breadth of data and the depth of information that can be provided by the lived experience of the staff engaging in the intervention. The consistent application of these outcomes can further our understanding of whether and how the implementation process was successful, what made it successful and which intervention components and implementation strategies had the strongest impact.

Our approach to operationalizing success outcomes highlighted several key lessons and useful steps to consider when measuring success. First, selecting and consulting key theoretical frameworks such as the Implementation Outcomes framework and STaRI guidelines was an essential first step to ensure we were capturing relevant dimensions. This approach is consistent with implementation science principles to use theory in project design, conduct, analysis and evaluation.

Second, we needed to define each success outcome within the context of our specific intervention (ADAPT CP) and research design (evaluating implementation strategies within a cluster RCT) before moving on to defining measures. This entailed deciding on the unit of analysis (individual or organizational level) for which success is most meaningful. We decided to address both, by gathering objective data on organizational outcomes, as well as at the individual level via staff perceptions of organization outcomes and of their own individual outcomes.

Third, we needed to define the elements (intervention, resources and implementation strategies) that were addressed under each success outcome. As shown in Table 2, mapping how definitions were applied across the cluster RCT, the timing of measurements and the existing sources of measurement. Systematically mapping our defined success outcomes and elements against existing measures was particularly helpful in ensuring confidence we had fully captured all relevant aspects of success.

Fourth, we needed to be flexible in combining psychometrically proven measures (to ensure measurement reliability and validity and allow comparison with other literature) and creating our own quantitative and qualitative questions and checklists (to ensure we had covered all theoretically important and contextual aspects).

Fifth, our guiding frameworks highlighted the need to provide a rich description of context to assist understanding of the interplay between the intervention, the providers, and the service, particularly if seeking to assess whether the implementation strategies can be transposed to other settings or will require adaption. Thus, we were careful to include a longitudinal qualitative component to measurement of success.

**Figure 1:**
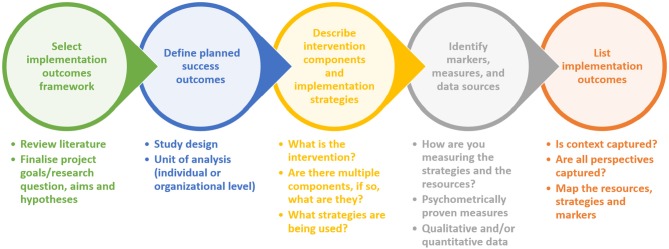
Targeting definable implementation outcomes. A conceptual approach to enable comparison of the effectiveness of implementation strategies and intervention components in a cluster randomized trial.

We have illustrated how we targeted defining implementation success and applying it to a cluster RCT focused on answering implementation effectiveness, in Figure 1, showing the concepts and questions that may be helpful to researchers in evaluating the success of the intervention being implemented as well as the implementation itself.

An ongoing challenge for the ADAPT Cluster RCT will be balancing the process of obtaining data for measurement of success without unduly influencing the process of the intervention and implementation, or overburdening participants with data collection throughout the implementation process.

Finally, the process of decision-making about success outcomes early in the process was recognized as a key part of the ADAPT research program, requiring regular iterative review and openness to flexibility, noting that assumed “essential” intervention components or implementation strategies may not turn out to be so. This work highlights the need to carefully consider multi-faceted success and end user perspectives as a key component in the evaluation of complex interventions and their implementation.

## Limitations

This paper provides an example of how in planning for a large cluster randomized trial with implementation success as the focus of the research questions, we tackled the planned collection and mapping of data to an implementation outcomes framework. We do not present the results of the cluster randomized trial, rather provide guidance to researchers and health services about how to approach projects in this field. For this research project, we were able to manage data collection using the resources of the research team, however we do not address issues for those seeking ongoing evaluation of initiatives, although reporting set up within initial implementation may be sustainable within institutions. This aspect warrants further enquiry and recommendations for researchers and health services.

## Conclusion

We have described a conceptual approach to selecting and defining success outcomes and what issues arose in making these specific to the ADAPT Cluster RCT. We believe this approach provides guidance to researchers and program leaders in implementation science to carefully design studies that collect data for both intervention components and implementation strategies. We contend that these findings have a range of implications for other implementation researchers, health services staff and policy makers, including the need to think about multi-faceted success, and grounding definitions in theoretical frameworks. We also note the importance of thorough review of the range of available measures to identify those that best fit with the implementation strategy and intervention context, and not impacting intervention or implementation burden by extensive additional data collection.

## Data Availability Statement

No trial data is presented in the paper. The study protocol for the cluster RCT used as an example for this paper is published, Butow et al. (19).

## Ethics Statement

The Cluster RCT used as an example for this methodological paper was approved by the Sydney Local Health District (RPAH Zone) Human Research Ethics Committee, Protocol X16-0378 HREC/16/RPAH/522.

## Author Contributions

HS and LG wrote the first draft of the manuscript. NR and PBu made significant contributions to subsequent drafts. HS compiled revisions from authors and prepared the final draft of the manuscript for submission. All authors were involved in developing the content of the manuscript through their participation in the ADAPT Working Groups, conceptualizing the markers of success and mapping them to the ADAPT Cluster RCT design, contributed to making revisions and responding to reviewer feedback, read and approved the final manuscript.

### Conflict of Interest

The authors declare that the research was conducted in the absence of any commercial or financial relationships that could be construed as a potential conflict of interest.
